# A Candy Defect Detection Method Based on StyleGAN2 and Improved YOLOv7 for Imbalanced Data

**DOI:** 10.3390/foods13203343

**Published:** 2024-10-21

**Authors:** Xingyou Li, Sheng Xue, Zhenye Li, Xiaodong Fang, Tingting Zhu, Chao Ni

**Affiliations:** College of Mechanical and Electronic Engineering, Nanjing Forestry University, Nanjing 210037, China; lixingyou@njfu.edu.cn (X.L.); xuesheng@njfu.edu.cn (S.X.); zhenye@njfu.edu.cn (Z.L.); fangxiaodong@njfu.edu.cn (X.F.); tingtingzhu@njfu.edu.cn (T.Z.)

**Keywords:** computer vision, deep learning, defects detection, generative adversarial networks, YOLOv7

## Abstract

Quality management in the candy industry is a vital part of food quality management. Defective candies significantly affect subsequent packaging and consumption, impacting the efficiency of candy manufacturers and the consumer experience. However, challenges exist in candy defect detection on food production lines due to the small size of the targets and defects, as well as the difficulty of batch sampling defects from automated production lines. A high-precision candy defect detection method based on deep learning is proposed in this paper. Initially, pseudo-defective candy images are generated based on Style Generative Adversarial Network-v2 (StyleGAN2), thereby enhancing the authenticity of these synthetic defect images. Following the separation of the background based on the color characteristics of the defective candies on the conveyor belt, a GAN is utilized for negative sample data enhancement. This effectively reduces the impact of data imbalance between complete and defective candies on the model’s detection performance. Secondly, considering the challenges brought by the small size and random shape of candy defects to target detection, the efficient target detection method YOLOv7 is improved. The Spatial Pyramid Pooling Fast Cross Stage Partial Connection (SPPFCSPC) module, the C3C2 module, and the global attention mechanism are introduced to enhance feature extraction precision. The improved model achieves a 3.0% increase in recognition accuracy and a 3.7% increase in recall rate while supporting real-time recognition scenery. This method not only enhances the efficiency of food quality management but also promotes the application of computer vision and deep learning in industrial production.

## 1. Introduction

China has gradually emerged as the world’s largest producer and seller of candy, making the candy industry an indispensable part of the leisure food sector. At the same time, candy companies are increasingly focusing on the improvement in production technology and quality management [1]. However, during the production process, candies are prone to defects such as bulging, depressions, and fragmentation. In candy production quality management, the traditional reliance on the manual selection of defective candies often results in a high rate of missed inspections. Hard candies with bulges cannot be packaged according to fixed specifications, thereby reducing production efficiency. Concave or broken candies may contain sharp edges, posing a risk to consumer safety and negatively impacting taste. Therefore, improving the production technology and quality management in the candy production line through machine vision-based defect detection algorithms has significant implications for promoting both the leisure food industry and the broader food industry [2].

Traditional food processing and defect detection methods necessitate continuous monitoring by personnel to observe product variations, which may lead to significant human errors. In contrast, recent advancements in machine vision and deep learning technologies have equipped food processing and detection systems with the capability to perform real-time non-destructive testing [3]. This enhancement is facilitated through the integration of cameras, models, and algorithms, enabling a comprehensive analysis of visual data based on parameters such as shape, size, and color [4]. Teimouri et al. used charge-coupled device (CCD) cameras and Artificial Neural Network (ANN) to classify chicken meat, achieving 93% overall accuracy on a 0.2 m/s production line [5]. Fan et al. utilized a deep learning architecture based on Convolutional Neural Networks (CNNs) to detect defective apples on a four-line fruit sorting machine at a speed of five fruits per second, achieving an accuracy of 96.5% [6]. Zhu et al. employed Support Vector Machines (SVMs) with the YOLOv3 network to implement the binary classification of banana ripeness and reached an overall accuracy of 96.4% [4]. Truong et al. utilized the YOLOv5 algorithm for target detection to detect surface defects in hardwood flooring, accurately detecting two specific defects [7]. Wang et al. used an improved YOLOv5 combined with the Convolutional Block Attention Module (CBAM) to achieve anomaly detection for closed vibrating screens [8]. These examples illustrate that the effective integration of deep learning and machine vision can address a multitude of practical problems. Consequently, the application of deep learning-based neural networks combined with machine vision to achieve candy defect detection appears promising.

However, several challenges arise when combining machine vision and deep learning techniques to solve the problem presented in this paper. Traditional target detection algorithms based on deep learning require a large number of positive and negative candy samples for input training during grid training [9]. These algorithms perform poorly when trained with imbalanced samples, and the detection speed for small target samples does not meet the requirements of automated assembly lines. In the case presented in this paper, only 76 photos were captured, containing 682 complete samples and 206 defect samples. Such an imbalanced training sample size could lead to model overfitting, making it challenging to achieve optimal detection performance. In the actual candy production line that requires upgrading, gathering a sufficient quantity and variety of defective samples is impractical.

When the positive and negative samples are unbalanced in target detection, the recognition accuracy can be improved by changing the sampling method and generating samples. Zhang et al. used the anchor point optimization module to determine the confidence score of the anchor point in the pipeline detection and employed thresholds to eliminate negative anchor points that are prone to occurring [10]. Chou et al. designed a defect coffee bean detection scheme based on deep learning and proposed a data-enhanced Generative Adversarial Network (GAN) structure for automatic labeling and data augmentation [11]. Guo et al. designed a data enhancement method combining a deep convolution generative adversarial network and rigid transformation, which effectively solved the misjudgment problem of finding defective jujube and healthy jujube. Currently, using data augmentation techniques to increase the number and diversity of imbalanced samples is an efficient method [12].

In this paper, foreground–background separation is initially employed to separate the defective candy targets from the conveyor belt background. Subsequently, Style Generative Adversarial Network-v2 (StyleGAN2) is used to generate 900 labeled negative samples from the 162 defective candy samples. These generated defective candy samples are then merged with original real background images. Finally, an improved YOLOv7 model is proposed, which uses the generated synthetic data for training and conducts inference with a faster speed to fit the requirements of the industry environment.

The main contributions of this paper can be summarized as follows:

(1) To address the issue of irrelevant information occupying a significant portion of space during the GAN-based generation of synthetic defective candy samples, we employed the foreground–background separation algorithm to isolate the labeled defective candy samples from the captured images one by one.
(2) To mitigate the impact of data imbalance between complete and defective candy samples on model accuracy, based on StyleGAN2, we employed the isolated defective candy samples to generate synthetic images.
(3) To enhance the performance of the defect detection model, we improved the YOLOv7 object detection model and integrated the global attention mechanism, thereby enabling the precise identification of small defects in candy samples.

## 2. Materials and Methods

### 2.1. Data Acquisition

**Figure 1:**
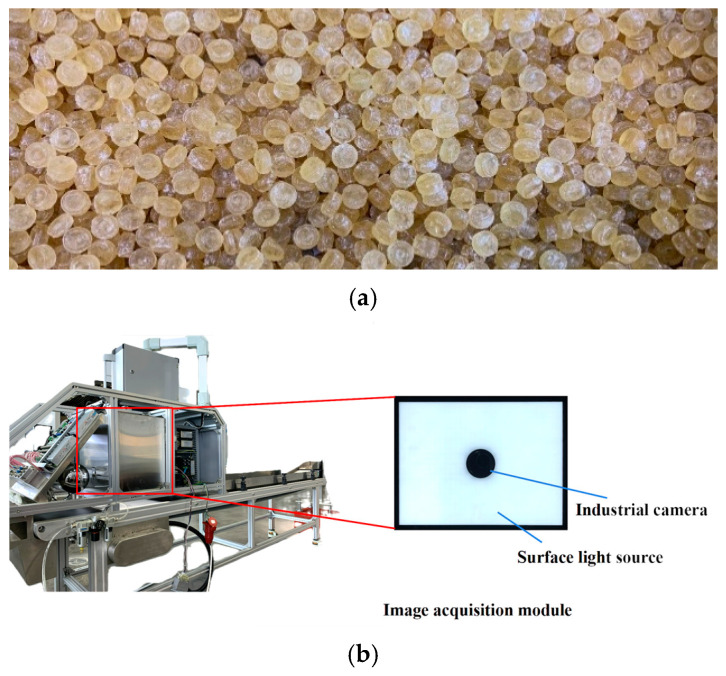

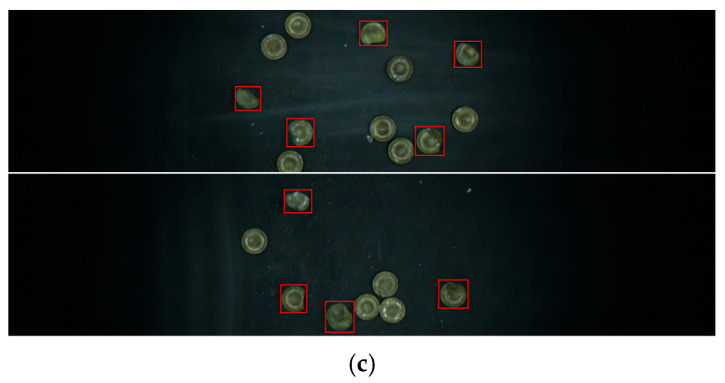
Candy sorting equipment and candy samples. (**a**) Candies produced by industrial manufacturing. (**b**) Overall side view of the sorting equipment and its image acquisition module. (**c**) Two candy sample images captured by the area scan industrial camera used in this paper (defective candy samples are marked in red boxes).

The data used in this paper were obtained from Nantong Wealth Machinery Technical Co., Ltd., located in Nantong, Jiangsu, China. This company specializes in the design and manufacture of various types of confectionery production and packaging equipment. The candy surface images obtained in this paper were captured by an area scan industrial camera installed above the candy sorting equipment. This camera captures images with the size of 2376 × 584 pixels, utilizing a pixel matrix for exposure at a rate of 10 frames per second. The captured image data are transmitted to a vision workstation, where candy defect detection and recognition algorithms are executed. The candies used in this paper were light brown, semi-transparent, round hard candies produced by the company, with a diameter of 1.2 cm and a weight of 2 g per piece. Due to limitations in the candy production line, the color of the candy images captured by the industrial camera may vary. Therefore, the defect detection model in this study focused exclusively on shape defects. According to the company’s production standards, a candy is classified as defective if the top-view area deviates from the standard by more than 10%, and defective candies must be detected and subsequently removed during the production process. Shape defects, such as bulging, indentation, and breakage, are present in the candies, often exhibiting subtle features and a wide range of variations, which pose significant challenges for accurate detection and recognition. Considering the imbalance between defective and complete candy samples in the industrial production processes, it is necessary to increase the quantity and defect types of defective candy samples to enhance the performance of the candy defect detection algorithm based on the target detection network. The candy sorting equipment and some defective candy samples are shown in Figure 1.

### 2.2. Manual Data Augmentation

**Figure 2:**
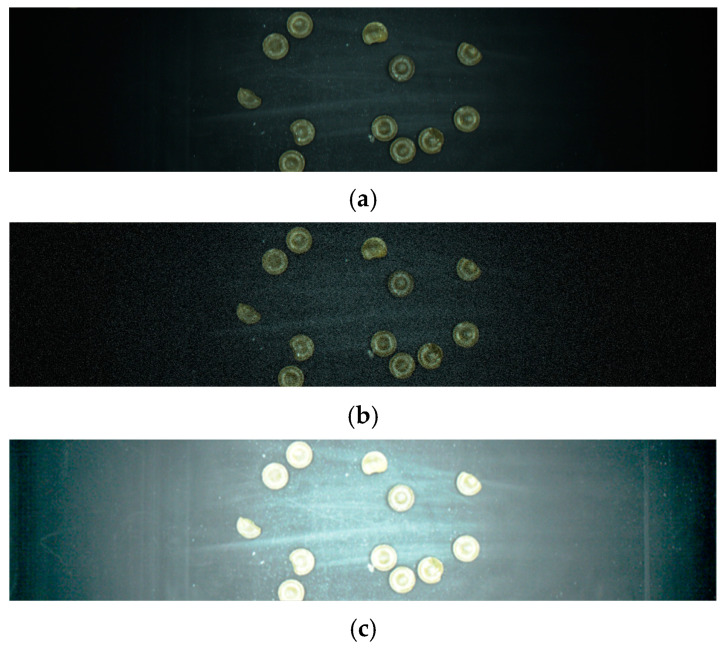
Manual data augmentation used in this paper. (**a**) Original candy images. (**b**) Noise injection. (**c**) Histogram equalization.

In this section, we describe manual data augmentation methods such as noise injection and histogram equalization [13,14]. As previously mentioned, the original dataset in this paper included 682 complete samples and 206 defective samples, showing a substantial imbalance between the two types of samples. Applying a deep learning model directly may lead to overfitting. To mitigate this issue, we artificially augmented the dataset through data augmentation to reduce the disparity in the number of the two types of samples. Furthermore, to maintain efficient training speeds for the candy defect detection model after data augmentation, and to minimize the labeling effort for the augmented samples, this paper adopted manual data augmentation methods that preserve the original image size and retain the existing label information. All manual data augmentation examples are shown in Figure 2.

In addition, the defective candies separated from the foreground and background will be automatically labeled with negative samples after data augmentation. There is no need to label generated pseudo-sample images one by one when training the detection and recognition algorithm, which greatly reduces the workload of manually labeling defective candies.

#### 2.2.1. Noise Injection

In order to simulate the noise caused by the long-term operation of area scan industrial cameras or the interaction of various components in the actual candy production process, we injected Gaussian noise into the collected candy sample images. In the Gaussian noise model, the image intensity was modified by a random Gaussian sampling distribution formula. The injection of noise increases the difficulty of sample feature learning, thereby enhancing the robustness of the candy defect detection model.

#### 2.2.2. Histogram Equalization

The image histogram reflects the frequency distribution of image color values. Due to the similarity between the color of the captured candies and the color of the conveyor belt, the surface features of the candies may not be displayed clearly. In this paper, we converted the sample images from the RGB color space to HSV and enhanced the contrast of the images. We performed normalization on the image histogram as follows:

(1) $$H\left(g_{k}\right)=\frac{n_{k}}{n},k=0,1,2\ldots ,L-1.$$

where $g_{k}$ represents the k gray level of the image, n represents the total number of pixels in the image, L represents the gray level of the image, and $n_{k}$ represents the number of pixels in the image in the condition of $g_{k}$. The cumulative distribution function maps $g_{k}$ to $C_{k}$ and obtains the new histogram:

(2) $$C_{k}=\sum_{i=0}^{k}\frac{n_{i}}{n}=\sum_{i=0}^{k}H\left(g_{k}\right),\;k=0,1,2\ldots ,L-1.$$

### 2.3. Generative Adversarial Network Data Augmentation

In this section, we performed foreground–background separation on the captured candy sample images and used StyleGAN2 based on the characteristics of imbalanced, dark color, and small size of candy defect sample data.

#### 2.3.1. Foreground–Background Separation

Due to the limited number of pixels occupied by a single candy sample in the images captured by the area scan industrial camera, the features of candy defects are not prominent. If generative adversarial network data augmentation is directly applied to the captured images, the generated pseudo-sample images cannot depict the features of defective candies. Moreover, the large area of the conveyor belt background consumes considerable computational resources during the data augmentation process. Therefore, we performed foreground–background separation on the images captured from the candy defect detection industrial assembly line. Subsequently, we extracted defect candy samples one by one and utilized them for generative adversarial network data augmentation.

**Figure 3:**
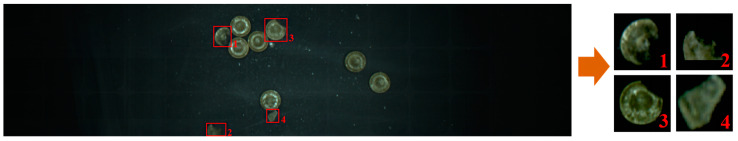
Foreground and background separation process (defective candy samples are marked in 1–4).

The foreground–background separation operation is challenging due to noise such as conveyor belt reflections present in the captured candy sample images and overlapping edges between some candies. In this paper, we integrated methods including Mean-Shift color smoothing, Canny edge detection, erosion dilation, and find-contour to systematically separate defective candy samples from the captured images [15,16,17,18]. The prospect of foreground–background separation for defective candy samples is shown in Figure 3.

#### 2.3.2. Defective Candy Data Augmentation

The significance and challenge of defective candy data augmentation is that there are only 682 complete samples and 206 defect samples obtained, which is greatly imbalanced with the complete candy sample data, and the shape and size of candy defects are quite different. In this paper, we used StyleGAN2 to generate clearer pseudo-sample images of defective candies.

GAN learns and trains on the distribution of the dataset through the generator G and the discriminator D. The generator G maps the input latent vector z∈Z (usually drawn by Gaussian distribution) to the generated pseudo-sample G(z) [19]. The discriminator D learns to distinguish between the generated pseudo-sample G(z) and the real sample x. The objective of the GAN network is to minimize the joint loss function of the generator and the discriminator, which is usually expressed as follows:

(3) $$L_{G}=E_{z\sim P_{z}\left(z\right)}\left[f_{G}\left(-D\left(G\left(z\right)\right)\right)\right]$$

(4) $$L_{D}=E_{x\sim P_{D}\left(x\right)}\left[f_{D}\left(-D\left(x\right)\right)\right]+E_{z\sim P_{z}\left(z\right)}\left[f_{G}\left(D\left(G\left(z\right)\right)\right)\right]$$

where $L_{G}$ represents the loss value of the generator G, $L_{D}$ represents the loss value of the discriminator D, and $f_{G}$ and $f_{D}$ are loss functions.

**Figure 4:**
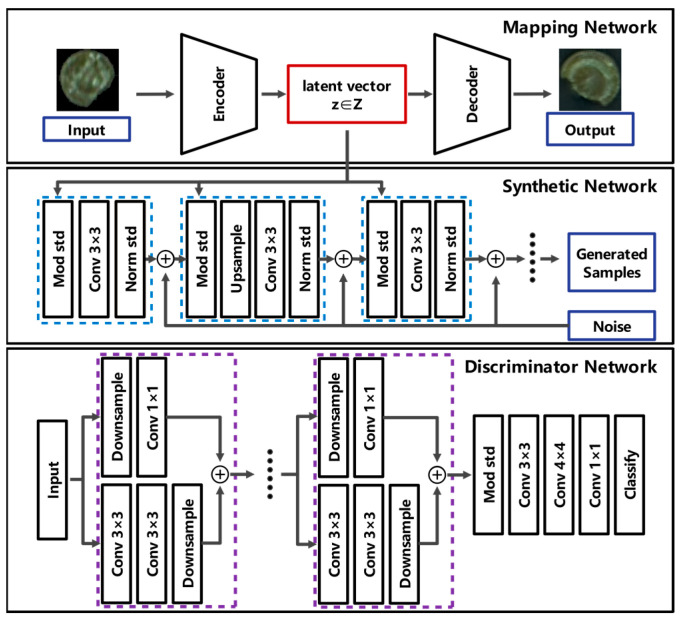
The network structure of StyleGAN2.

StyleGAN2 mainly consists of three components: the mapping network, the synthesis network, and the discriminator network. The mapping network of StyleGAN2 encodes the input vector into an intermediate vector, which is then passed to the generation network to obtain the control vector. This allows different elements of the control vector to control different style features, effectively eliminating feature entanglement among control vectors [20]. However, due to the unconditional generation characteristics of GANs, the model’s performance in data augmentation is limited, often resulting in redundant synthetic samples. Consequently, GANs encounter difficulties when tasked with generating a large number of pseudo-sample images. To enhance the subtle defect features of the pseudo-samples, noise is introduced into the synthesis network of StyleGAN2, introducing stochastic variations that allow for the generation of pseudo-sample images with defects in varying shapes, locations, and sizes. This incorporation of noise enhances the realism and natural appearance of the synthetic candy samples. The network structure of StyleGAN2 is shown in Figure 4. StyleGAN2 can generate defect candy sample images with distinctive features and has a good generalization, which improves the performance of the data augmentation model.

### 2.4. Defective Candy Detection and Recognition

This paper adopted an improved YOLOv7 model for candy defect detection. YOLOv7 leverages techniques such as deep supervision, dynamic label allocation, and guided label assignment to improve the model’s performance. However, shortcomings exist in the structure of YOLOv7. Firstly, YOLOv7 employs the Spatial Pyramid Pooling Cross Stage Partial Connection (SPPCSPC) module to adapt to detection targets of different sizes, but it may ignore unmatched targets in different pooling channels, which leads to feature loss and affects the accuracy of the model. Secondly, YOLOv7 utilizes the efficient layer aggregation network (ELAN) module to precisely extract features from the input feature map; however, it incorporates more convolutional modules and residual connections, resulting in an increased computational complexity and decreased calculation speed. These issues impact the performance of candy defect detection, particularly in scenarios involving numerous small-sized, unevenly distributed, and inconspicuous candy defects as observed in this paper, where YOLOv7 fails to achieve high-speed and high-accuracy detection and recognition.

#### 2.4.1. Improved YOLOv7 Detection Algorithm

**Figure 5:**
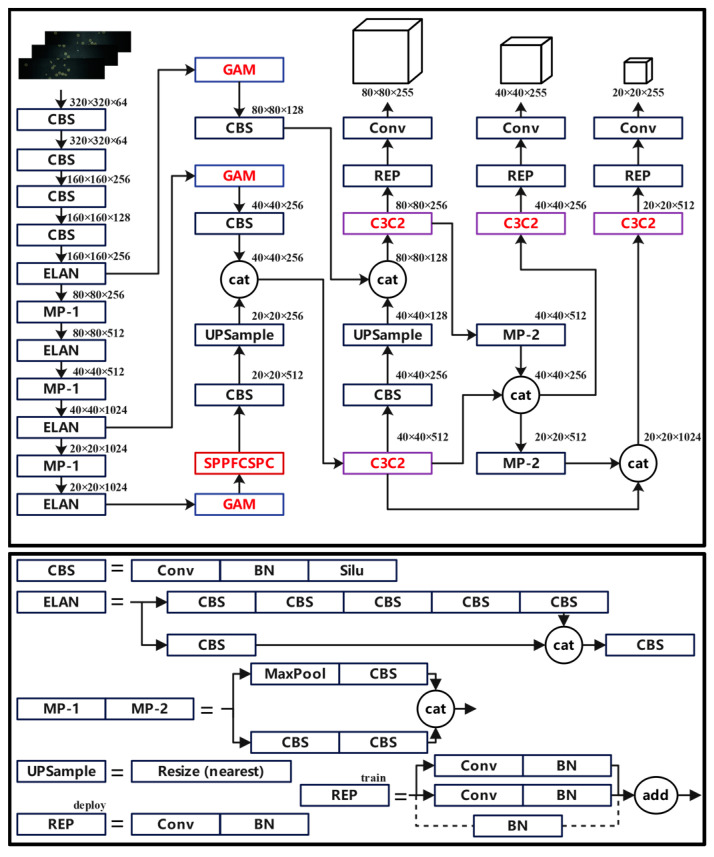
The network structure of the improved YOLOv7 (the modules improved in this paper are highlighted in red).

To overcome the shortcomings of YOLOv7 in small object detection like candy defect detection, this paper introduced three primary improvements to its structure. Firstly, we replaced the SPPCSPC module with the SPPFCSPC module to reduce the complexity of the model and improve the detection accuracy of small target candy defects. Secondly, we introduced the C3C2 module to replace the ELAN module following the concatenation operation, which improves the speed and accuracy of candy defect feature extraction. Thirdly, we incorporated the widely adopted Global Attention Mechanism (GAM) to improve the model’s capability in extracting critical feature information in the process of small target detection. The improved YOLOv7 structure is shown in Figure 5.

#### 2.4.2. SPPFCSPC Module

**Figure 6:**
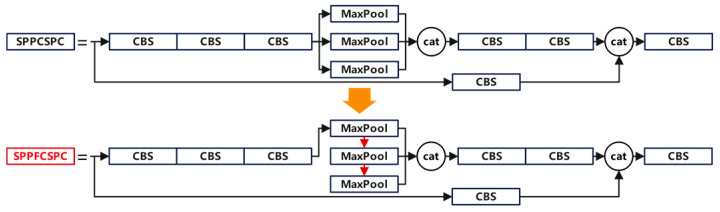
The comparison between the SPPFCSPC module and the SPPCSPC module.

The SPPFCSPC module uses pooling layers with a kernel size of 5 to replace the pooling layers with kernel sizes of 5,9, and 13 in the SPPCSPC module [21]. In this module, the output of each pooling layer is passed to the next pooling layer and the final connection layer, which reduces the model’s complexity and calculation time. The comparison between the SPPFCSPC module and the SPPCSPC module is shown in Figure 6.

#### 2.4.3. C3C2 Module

In the process of candy defect identification and detection, due to the random generation of candy defects, there may be great differences in size and shape. These factors pose great difficulties to the feature extraction process of candy defects in the ELAN module of YOLOv7, resulting in a decrease in recognition accuracy. Additionally, the convolutional modules and residual connections in the ELAN structure of the model also increase the computational complexity and reduce the speed of defect candy identification and detection. To improve the above shortcomings, the C3C2 module is used to replace the ELAN module following the concatenation operation.

**Figure 7:**
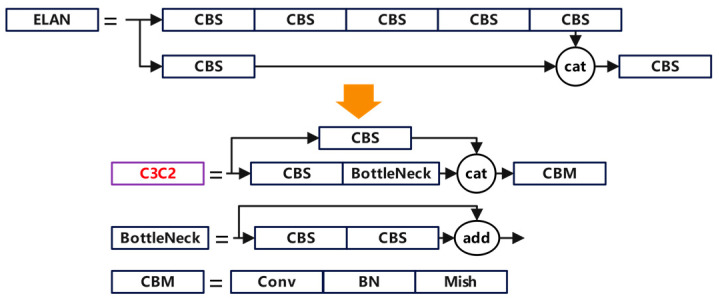
The comparison between the C3C2 module and the ELAN module.

The C3C2 module simplifies the residual branch convolution module of the C3 module into a simple convolution structure and omits the BN layer and the activation function layer, reducing the calculation amount of the model. In addition, the C3C2 module replaces the activation function of the final convolution module from the SILU function to the MISH function. This change introduces non-linear transformation when the model inputs into the convolution module after the concatenation operation, aiding the candy defect identification and detection model to have a better overfitting capability [22]. The comparison between the C3C2 module and the ELAN module is shown in Figure 7.

#### 2.4.4. Global Attention Mechanism

**Figure 8:**
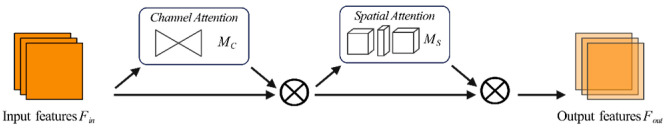
The structure of the global attention mechanism.

In the detection process of small objects such as candy defects, if attention is focused on regions of interest, more favorable key features will be extracted. The Global Attention Mechanism (GAM) improves the accuracy of model detection and recognition by selectively attending to desired parts in both channels and spatial dimensions [23]. The GAM structure is shown in Figure 8, which uses the sequential channel spatial attention mechanism from CBAM and makes improvements to its sub-modules. The GAM state expression is as follows:

(5) $$F_{out}=M_{s}\left[M_{c}\left(F_{in}\right)⨂F_{in}\right]⨂\left[M_{c}\left(F_{in}\right)⨂F_{in}\right]$$

where $F_{in}$ and $F_{out}$ represent the input and output features, respectively; and $M_{c}$ and $M_{s}$ represent channel and spatial attention, respectively.

The channel attention sub-module retains feature information across three dimensions, where the multi-layer perceptron is used to amplify cross-dimensional channel spatial correlations. The spatial attention sub-module achieves spatial information fusion through two convolution layers. In this paper, by adding three GAM modules, the loss of candy feature information was reduced, and the global interactive features were amplified, which improved the speed and accuracy of the model’s detection and recognition of candy defects.

### 2.5. Training Settings

StyleGAN2 and the candy defect recognition detection algorithm based on improved YOLOv7 were deployed and trained on PyCharm (version 2022.1). The platform configuration used for training in this experiment was as follows: Windows 10 Professional operating system, Intel(R) Core(TM) i9-9900K (3.60 GHz processor), and NVIDIA GeForce RTX2080 Ti graphics card. During the data augmentation process, the training and testing ratio of StyleGAN2 was set to 10:0, where the model did not require a separate testing set. All 76 captured candy images were utilized for model training. In the candy defect detection and recognition process, the training and testing ratio of the improved YOLOv7 was approximately 5:1. Additionally, the dataset provided bounding box annotations indicating the category of candies in each image.

### 2.6. Performance Evaluation

We introduced the Fréchet Inception Distance (FID), which is used to measure the similarity between generated images and real images, the Learning Perceptual Image Patch Similarity (LPIPS), and the Multi-Scale Structural Similarity Index (MS-SSIM), which are used to evaluate the visual quality and structural features of generated images. Additionally, we introduced average precision (AP), which is a metric of the performance of the candy defect detection and recognition network.

#### 2.6.1. Fréchet Inception Distance

Based on the Fréchet distance, Fréchet Inception Distance (FID) uses the pre-trained inception network to extract image features, maps the images from pixel space to feature space, and calculates the difference between the generated samples and real samples in terms of their means and covariance in this space [24]. Given the feature mean $\mu_{g}$ of the generated defective candy samples and the feature mean $\mu_{r}$ of the real defective candy samples, the expression of FID is:

$$FID={\left\|\mu_{r}-\mu_{g}\right\|}^{2}+Tr\left(C_{r}+C_{g}-2{\left(C_{r}C_{g}\right)}^{\frac{1}{2}}\right)$$

where $C_{r}$ and $C_{g}$ are the covariance matrices of the real defective candy samples and the generated defective candy samples, respectively, and $Tr\left(\cdot \right)$ represents the trace of the matrix.

#### 2.6.2. Learning Perceptual Image Patch Similarity

Learning Perceptual Image Patch Similarity (LPIPS) maps images into the feature space through a pre-trained deep convolutional neural network and measures the perceptual similarity between images by comparing the distances between these feature representations [25]. Given the generated defective candy $x_{g}$ and the real defective candy $y_{r}$, the expression of LPIPS is:

(6) $$LPIPS\left(x_{g},y_{r}\right)=\frac{1}{M}\sum_{j=1}^{M}\sum_{i=1}^{N}\omega_{i}\cdot {\left\|\phi_{i}\left(x_{g_{j}}\right)-\phi_{i}\left(y_{r_{j}}\right)\right\|}_{2}$$

where N represents the number of features extracted in the network, $\omega_{i}$ represents the weight of the i-th layer, and $\phi_{i}\left(\cdot \right)$ represents the deep convolutional neural network used to extract image features.

#### 2.6.3. Multi-Scale Structural Similarity Index

Multi-Scale Structural Similarity Index (MS-SSIM), based on the traditional structural similarity index (SSIM), considers the structural similarity at multiple scales, which is more convenient and advantageous in capturing image details [26]. Given the generated defective candy $x_{g}$ and the real defective candy $y_{r}$, the expressions of MS-SSIM are as follows:

(7) $$SSIM\left(x_{g},y_{r}\right)=\frac{\left(2\mu_{x_{g}}\mu_{y_{r}}+C_{1}\right)\left(2\sigma_{x_{g}y_{r}}+C_{2}\right)}{\left(\mu_{x_{g}}^{2}+\mu_{y_{r}}^{2}+C_{1}\right)\left(\sigma_{x_{g}}^{2}+\sigma_{y_{r}}^{2}+C_{2}\right)}$$

(8) $$MS-SSIM\left(x_{g},y_{r}\right)=\frac{1}{N}\sum_{i=1}^{N}{SSIM}_{i}\left(x_{g},y_{r}\right)$$

where $\mu_{x_{g}}$ and $\mu_{y_{r}}$ represent the mean of the images $x_{g}$ and $y_{r}$, respectively; $\mu_{x_{g}}^{2}$ and $\mu_{y_{r}}^{2}$ represent the variance of the images $x_{g}$ and $y_{r}$, respectively; $\sigma_{x_{g}y_{r}}$ represents the covariance between images $x_{g}$ and $y_{r}$; $C_{1}$ and $C_{2}$ are constants added for stability; N represents the total number of windows; and ${SSIM}_{i}\left(x_{g},y_{r}\right)$ represents the SSIM value at scale i.

#### 2.6.4. Average Precision

Average precision (AP) measures the accuracy of candy defect detection and recognition. We used average precision as the model performance evaluation criterion [27]. The relevant parameters are calculated as follows:

(9) $$Precision=\frac{TP}{TP+FP}$$

(10) $$Recall=\frac{TP}{TP+FN}$$

(11) $$AP=\int_{0}^{1}P\left(R\right)dR$$

where TP represents true-positive candy defects, FP represents false-positive candy defects, and FN represents false-negative candy defects. P and R denote precision and recall, respectively.

### 2.7. Data Analysis and Application Deployment

We divided the entire candy defect detection process into two stages: data analysis and application deployment.

#### 2.7.1. Data Analysis

Step 1: Obtain color images containing defective candies through the candy production line detection equipment.

Step 2: Perform manual data augmentation on the captured images through noise injection and histogram equalization.

Step 3: Separate the foreground and background of the color images, and selectively filter defective candy samples.

In the process of foreground–background image separation, mean-shift color smoothing can filter out the interference noise in candy sample images and achieve the adaptive smoothing of locally important edge information. Canny edge detection segments the image through high and low thresholds to obtain edge images.

Step 4: Perform forward and backward propagation in StyleGAN2 until the model converges.

StyleGAN2 model optimization: The defective candy samples $x_{Candy}\in N^{h\times w\times c}\;$separated in step 3 are used to perform data augmentation, where h, w, and c represent its height, width, and color features, respectively. During the forward propagation process, StyleGAN2 generates random latent vectors Z and utilizes mapping networks to transform them into intermediate latent vectors W and estimate the mean of intermediate latent vectors μ. The best Generative Adversarial Network is obtained by optimizing μ. In addition, to facilitate the latent vector search, noise $n_{i}\in R^{r_{i}\times r_{i}}$, where $r_{i}\in \left\{1024,512,\ldots ,8\right\}$, is added to the backpropagation to optimize the synthesis process of defective candies [28]. The pseudocode of StyleGAN2 latent vector W and noise N generation algorithm is shown inAlgorithm 1; the pseudocode of the StyleGAN2 generator $G_{Candy}$ generation algorithm is shown in Algorithm 2.

Step 5: Use the best StyleGAN2 to generate defective candy pseudo-samples.

Step 6: Fuse the generated single defective candy pseudo-samples into the original images.

Step 7: Select 65 original images, 8 images with manual data augmentation, and 27 images with StyleGAN2 data augmentation as the training set for candy defect detection and recognition. Additionally, select 11 original images, 2 images with manual data augmentation, and 7 images with StyleGAN2 data augmentation as the testing set for candy defect detection and recognition.

Step 8: Utilize the LabelView platform to manually annotate the training set and the test set. The training set consists of 1336 candy samples, including 708 defective candy samples. The test set consists of 253 candy samples, including 157 defective candy samples.

Step 9: Perform forward and backward propagation on the improved YOLOv7 algorithm to obtain the best-performing candy defect detection and recognition model.
**Algorithm 1.** StyleGAN2 latent vector and noise generation algorithm pseudocode.StyleGAN2 latent vector W noise N
    Input: Candy defective sample images $x_{Candy}$ and StyleGAN2 generator model          $G_{Candy}$

    Output: Latent vector w and set of noise maps $n_{i}$,i∈0,1,…,17

    1: $z=\left\{z_{1},z_{2},\ldots {,z}_{\alpha }\right\}\leftarrow \left\{0,1,\ldots ,\alpha \right\}$

    2: $w=\left\{w_{1},w_{2},\ldots {,w}_{\alpha }\right\}\leftarrow G\left(z\right)$

    3: $\mu \leftarrow \frac{\sum_{i}\left(w_{i}\right)}{N}$

    4: $\left\{n_{1},n_{2},\ldots {,n}_{i}\right\}\leftarrow \left\{R^{r_{1}\times r_{1}},R^{r_{2}\times r_{2}},\ldots ,R^{r_{i}\times r_{i}}\right\}$

    5: While not converge do

    6: $n_{i,j}\leftarrow Downsample\left(n_{i},0.5\right),0\leq j<i,j\epsilon N$

    7: $L\leftarrow {\left(\frac{1}{r_{i,j}^{2}}\sum_{x,y}n_{i,j}\left(x,y\right)n_{i,j}\left(x-1,y\right)\right)}^{2}+{\left(\frac{1}{r_{i,j}^{2}}\sum_{x,y}n_{i,j}\left(x,y\right)n_{i,j}\left(x,y-1\right)\right)}^{2}$

    8: ∇w←dL/dw

    9: While *i* in $\left(0,1,\ldots ,17\right)$ do

    10: $\nabla n_{i}\leftarrow dL/dn_{i}$

    11: $n_{i}=n_{i}-{\nabla n}_{i}$

    12: end while

    13: w=w−∇w

    14: end while

    15: return $w,n_{1},n_{2},\ldots ,n_{i}$


**Algorithm 2.** StyleGAN2 generator generation algorithm pseudocode.StyleGAN2 adapted generator $G_{candy}$
    Input: Candy defective sample images $x_{Candy}$, its corresponding closest latent     vector w, and StyleGAN2 generator G

    Output: StyleGAN2 adapted generator $G_{candy}$

    1: $G_{candy}\leftarrow G$

    2: $W\leftarrow weights\left(G_{candy}\right)$

    3: While not converging do

    4: $x_{Candy}\leftarrow G_{candy}\left(z\right)$

    5: ∇g←dL/dw

    6: $G_{candy}=G_{candy}+\nabla g$

    7: ∇w←dL/dw

    8: end while

    9: return $G_{candy}$


#### 2.7.2. Application Deployment

Step 10: Deploy the candy defect detection and recognition model to the candy production and inspection equipment through the Linux platform.

Step 11: Debug the equipment platform to ensure that the timings of conveyor belt transportation, camera acquisition, algorithm recognition, and spray valve switching are matched.

In actual production, the hardware component of the candy defect detection and identification system is manufactured and supplied by Nantong Wealth Machinery Technical Co., Ltd. The conveyor belt operates at a speed of 2.5 m/s, transporting the candies to the defect detection module. The area scan industrial camera captures images of the candy samples at a rate of 10 frames per second. The computer executes the deployed defect recognition model to analyze the images and subsequently converts the recognition results and coordinate information into the pulse states for the spray valves in the defect removal system at the end of the conveyor belt. As the candies reach the end of the conveyor belt, complete candies fall into the candy collection box, while defective candies have their flight trajectories altered by the airflow from the spray valves and fall into the defective candy collection box.

## 3. Results

In this section, we describe the experimental results. We evaluate the performance of StyleGAN2 and the candy defect recognition detection network proposed in this paper.

### 3.1. Generated Samples

This section discusses the performance of the different categories of GAN models in generating synthetic samples. We used 206 defective candies separated from the foreground and background to train different GAN models. Deep Convolutional Generative Adversarial Networks (DCGANs) apply convolutional neural network techniques to GAN networks [29]. Wasserstein Generative Adversarial Networks (WGANs) use Wasserstein distance as a way to measure the spatial data of generated data and training samples [30]. Big Generative Adversarial Networks (BigGANs) can generate high-resolution pseudo-images [31]. StyleGAN uses style to influence the visual features of the generated images [32]. The experimental comparison results between StyleGAN2 and several common GAN networks are shown in Table 1.

**Figure 9:**
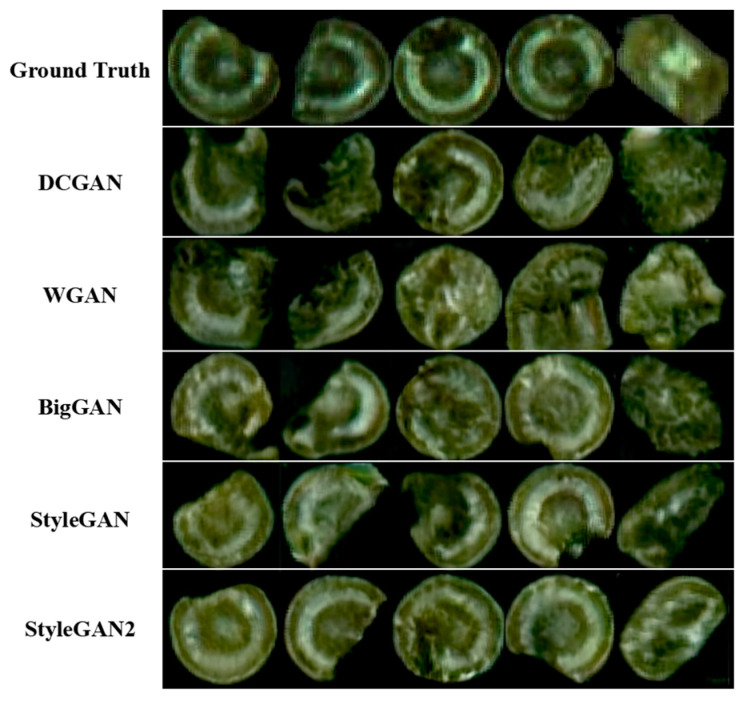
Sample generation results of different GAN methods by using all the original defective candy images.

Compared to other categories of GAN models, StyleGAN2 shows the best performance in both the LPIPS and MS-SSIM dimensions. The images generated by StyleGAN2 and several common GAN networks are shown in Figure 9. StyleGAN2 can generate clearer and more detailed images, providing stronger model generalization capabilities. It lays a solid foundation for candy defect identification and detection. In this paper, we generated 900 defective candy pseudo-samples through StyleGAN2, out of which 300 samples were selected to make 34 data augmentation images for training and test datasets.

### 3.2. Candy Defect Detection

To validate the performance of the candy defect recognition detection method proposed in this paper, we trained five advanced object recognition detection algorithms under the same dataset and parameter settings conditions, including Faster R-CNN, YOLOv5, YOLOX, YOLOv7, and the improved YOLOv7 proposed in this paper [33,34,35,36]. During the training phase, the parameters of these models were uniform and are shown in Table 2.

**Figure 10:**
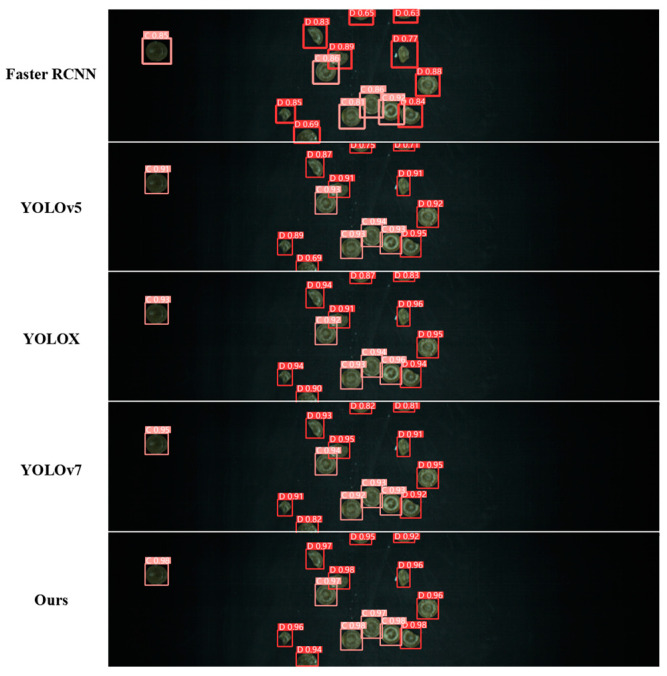
Detection results of different target models.

The precision, recall rate, and mean average precision of different detection models are shown in Table 3, where the subscript represents the selected Intersection Over Union (IOU) value. ${AP}_{0.5:0.95}$ represents the average of ten values when the threshold is set from 0.5 to 0.95 and the step size is 0.05. The improved YOLOv7 model achieved the best results in all indicators, with a precision of 0.981 and a recall of 0.962. The detection and recognition results of different algorithms are shown in Figure 10. Candy categories were predicted through different deep target detection models. The red boxes mark defective candies, and the pink boxes mark complete candies. The numbers next to the boxes represent the confidence levels of the model predictions.

To ensure the positive impact of the improvements to YOLOv7 presented in this paper, we conducted ablation experiments in terms of average precision, inference speed, and model size. The results of these experiments are shown in Table 4. Due to the significant improvement in speed and light-weighting of the candy defect detection and recognition model brought by the introduction of the SPPFCSPC module, we retained the SPPFCSPC module in the ablation experiments to reduce the workload of useless comparative experiments. It is evident that the integration of the SPPFCSPC module, C3C2 module, and global attention mechanism improved the performance of the original YOLOv7 model in the task of candy surface defect detection.

In summary, under the interference of close distance between candies and small defects, the defect detection network is not accurate enough in predicting the target, and the missed detection and false detection of the YOLOv7 model are serious. The introduction of SPPFCSPC, C3C2, and GAM attention mechanisms based on YOLOv7 can fully extract the characteristics of small defects in candies and improve the detection performance of the detection network for defective candies.

Since the model proposed in this paper exhibited the best performance, we exclusively employed this algorithm to verify the data augmentation effect. To validate the performance of the manual data augmentation and StyleGAN2 proposed in this paper, we trained four different datasets under the same parameter settings and detection algorithms, including original images, original images–manual data augmentation, original images-StyleGAN2, and original images–manual data augmentation-StyleGAN2. The model recognition results under different data augmentation methods are shown in Table 5. Based on the relevant content shown in the table, we can draw the following conclusions. (1) Manual data augmentation has very limited improvement in the model accuracy and recall rate. We believe that manual data augmentation only enriches the amount of data and does not solve the problems of small defects and unclear features of candies. Even in the process of data augmentation, new problems such as candy image overlap will occur, resulting in performance degradation. (2) After adding pseudo-samples generated by StyleGAN2 to the training dataset, the accuracy of the model is significantly improved.

## 4. Discussion

According to the experimental results, the model proposed in this paper offers the following prominent contributions. (1) By employing mean-shift color smoothing, Canny edge detection, and threshold segmentation, the foreground and background of the labeled defective candy samples were effectively separated. This approach greatly enhanced the efficiency of data augmentation. (2) Compared to other GAN networks, StyleGAN2 can generate clearer and more complete images of synthetic defective candy samples. This approach not only improved the model’s detection performance but also reduced the workload of manually labeling defective candies. (3) By introducing the Spatial Pyramid Pooling Fast Cross Stage Partial Connection (SPPFCSPC) module, C3C2 module, and global attention mechanism, the improved YOLOv7 target detection model has a greater improvement in detection accuracy and recognition speed, making it more conducive to practical applications of candy production. This paper introduced a new method for defect detection in the food sector, addressing challenges related to imbalanced data. However, a limitation of our approach is that, when candies overlap and edge defects are not clearly discernible, the detection accuracy of the system still has significant room for improvement. Therefore, improving the performance of industrial cameras and further improving the detection accuracy and recognition speed of the model will be the focus of future efforts for our detection and recognition system.

Furthermore, to meet the production needs of candy manufacturers for products of varying shapes, new features can be explored to enhance the quality of the generated pseudo-samples and improve the accuracy of defect detection. Our method offers significant application potential and opportunities for enhancement, particularly in the detection of defects in irregularly shaped lollipops with plastic sticks. Additionally, this approach can be deployed in fields such as food defect detection involving imbalanced samples, food grading, and fruit sugar content prediction, demonstrating considerable potential for practical application.

## 5. Conclusions

This paper proposed a two-stage method to address defect detection in candy production processes. In the first stage, considering the data imbalance between complete and defective candy samples, we employed manual data augmentation and StyleGAN2 to generate synthetic defective candy samples. In the second stage, the YOLOv7 model was improved to achieve a higher detection accuracy and faster detection speed for candy defects, which enhanced its applicability for practical food production. This method achieved a 98.1% accuracy rate and a 95% recall rate for real-time defect detection, representing a 3% and 5% increase, respectively. In summary, this method provided a practical solution for defect detection tasks involving imbalanced and small sample data. However, the current limitation of this method lies in the small size of defect features and insufficient data, which may cause the GAN to generate a large number of irrelevant synthetic samples and use excessive computational resources. Future improvements in the detection system could focus on improving feature extraction capabilities and reducing the model size while maintaining both the accuracy and speed of feature learning.

## Figures and Tables

**Table 1:** LPIPS and MS-SSIM scores of defective candy images generated using different GAN networks.

Methods	FID ↓	LPIPS ↓	MS-SSIM ↑
DCGAN	33.9	0.178	0.724
WGAN	37.7	0.186	0.776
BigGAN	7.8	0.172	0.804
StyleGAN	4.1	0.147	0.731
StyleGAN2	2.6	0.113	0.893

**Table 2:** Training parameter settings.

Batch Size	Epochs	LWeight_Decay	Learning_Rate
16	600	0.0005	0.01

**Table 3:** Comparison of evaluation indicators of the different models.

Model	Precision	Recall	${mAP}_{@0.5}$	${mAP}_{@0.5:0.95}$
Faster R-CNN	0.833	0.791	0.853	0.603
YOLOv5	0.929	0.878	0.935	0.757
YOLOX	0.942	0.892	0.946	0.778
YOLOv7	0.951	0.926	0.955	0.769
Improved YOLOv7	0.981	0.962	0.977	0.806

**Table 4:** Results of the ablation studies.

Model	Precision	Recall	${mAP}_{@0.5:0.95}$	Speed (ms)	Size (Mb)
YOLOv7	0.951	0.925	0.769	10.9	73.1
YOLOv7-SPPFCSPC	0.950	0.928	0.771	7.6	48.8
YOLOv7-SPPFCSPC-C3C2	0.977	0.949	0.799	7.2	43.7
YOLOv7-SPPFCSPC-GAM	0.948	0.965	0.783	7.9	50.3
YOLOv7-SPPFCSPC-C3C2-GAM	0.981	0.962	0.806	7.3	43.1

**Table 5:** Indicators of models under different augmentation methods.

Methods	Precision	Recall	${mAP}_{@0.5}$	${mAP}_{@0.5:0.95}$
Original	0.923	0.916	0.945	0.748
Original + Manual data augmentation	0.927	0.915	0.939	0.752
Original + StyleGAN2	0.977	0.955	0.977	0.794
Original + Manual data augmentation + StyleGAN2	0.981	0.962	0.971	0.806

## Data Availability

The data presented in this study are available on request from the corresponding author due to the data are part of an ongoing study.
